# Heritability of ECG Biomarkers in the Netherlands Twin Registry Measured from Holter ECGs

**DOI:** 10.3389/fphys.2016.00154

**Published:** 2016-04-29

**Authors:** Emily C. Hodkinson, Melanie Neijts, Arash Sadrieh, Mohammad S. Imtiaz, Mathias Baumert, Rajesh N. Subbiah, Christopher S. Hayward, Dorret Boomsma, Gonneke Willemsen, Jamie I. Vandenberg, Adam P. Hill, Eco De Geus

**Affiliations:** ^1^Molecular Cardiology and Biophysics, Victor Chang Cardiac Research InstituteDarlinghurst, NSW, Australia; ^2^St Vincent's Clinical School, University of New South WalesSydney, NSW, Australia; ^3^Department of Biological Psychology, EMGO+ Institute, VU University and VU Medical CenterAmsterdam, Netherlands; ^4^School of Electrical and Electronic Engineering, University of AdelaideAdelaide, SA, Australia

**Keywords:** ECG, heritability, human genetics, twins, Holter electrocardiogram

## Abstract

**Introduction:** The resting ECG is the most commonly used tool to assess cardiac electrophysiology. Previous studies have estimated heritability of ECG parameters based on these snapshots of the cardiac electrical activity. In this study we set out to determine whether analysis of heart rate specific data from Holter ECGs allows more complete assessment of the heritability of ECG parameters.

**Methods and Results:** Holter ECGs were recorded from 221 twin pairs and analyzed using a multi-parameter beat binning approach. Heart rate dependent estimates of heritability for QRS duration, QT interval, T_peak_–T_end_ and T_height_ were calculated using structural equation modeling. QRS duration is largely determined by environmental factors whereas repolarization is primarily genetically determined. Heritability estimates of both QT interval and T_height_ were significantly higher when measured from Holter compared to resting ECGs and the heritability estimate of each was heart rate dependent. Analysis of the genetic contribution to correlation between repolarization parameters demonstrated that covariance of individual ECG parameters at different heart rates overlap but at each specific heart rate there was relatively little overlap in the genetic determinants of the different repolarization parameters.

**Conclusions:** Here we present the first study of heritability of repolarization parameters measured from Holter ECGs. Our data demonstrate that higher heritability can be estimated from the Holter than the resting ECG and reveals rate dependence in the genetic—environmental determinants of the ECG that has not previously been tractable. Future applications include deeper dissection of the ECG of participants with inherited cardiac electrical disease.

## Introduction

The electrocardiogram (ECG) is an extraordinarily useful non-invasive diagnostic tool that has been used in clinical electrophysiology for over a century (Fye, 1994). Understanding the genetic contribution to defining the waveforms on the ECG is critical in understanding heritability of disease as well as population variance in disease presentation. As a result many studies have addressed this question using twin studies (Russell et al., 1998; Carter et al., 2000; Mutikainen et al., 2009; Haarmark et al., 2011). All of these studies, however, used measures from resting ECGs. Typically, a resting ECG is recorded over ~10 s and gives a “snapshot” of the electrical activity of the heart, so precluding the analysis of any rate-dependence to heritability of ECG parameters.

As an alternative, recording cardiac electrical activity over a 24-h period using continuous ambulatory ECG (Holter) is a richer source of information with the potential to provide a more complete phenotypic picture (Coumel et al., 1994), and a more accurate estimate of heritability. No studies to date have evaluated heritability of ECG parameters related to repolarization from Holter ECGs. This is largely the result of the difficulties associated with significant inter-beat variability, signal noise, and manipulating and analyzing large data sets. Indeed, the usefulness of Holter recording in diseases of repolarization has been questioned (Mauriello et al., 2011). In this study we have negated these issues by using an extended selective beat binning approach (Badilini et al., 1999) based around features of the R wave—the most unambiguous feature of the ECG waveform—to generate averaged signals. By doing so we have been able to extract rate specific data from the Holter ECG and measure heritability of ECG parameters. Specifically, we tested: (1) whether heritability estimated from Holter ECGs is larger than heritability measured from resting ECGs, (2) the extent to which genetic determinates of depolarization and repolarization parameters are heart rate-dependent, and (3) Whether different genetic determinants are involved in the heritability of individual ECG parameters related to repolarization. Taken together, we test the hypothesis that analysis of rate dependent data from Holter ECGs allow for a more complete genetic dissection of cardiac electrophysiology than a resting ECG.

## Materials and methods

### Participants

Holter recordings were carried out on 442 participants—123 monozygotic (MZ) complete pairs and 98 dizygotic (DZ) complete twin pairs from the Netherlands Twins Registry (Neijts et al., 2014). Seventy nine participants were taking medication with the potential to alter the ECG (Supplemental Table 1) and were excluded from the main analysis. For comparison, twin correlations including participants on cardioactive medications are shown in Supplemental Table 2. The study was approved by the medical Ethics Committee of the VU University Medical Center Amsterdam and the Human Research Ethics Committee of the New South Wales Ministry of Health (Australia). All participants gave written consent before entering the study.

### Holter ECG recording procedure

Participants were visited in the morning at home or at the work location when this was deemed more convenient. They were fitted with the VU University Ambulatory Monitoring System (VU-AMS) device to record the electrocardiogram (ECG) continuously over a 24-h period; recording duration was 1440 ± 110 min (mean ± SD, *n* = 442), using seven disposable, pregelled Ag/AgCl electrodes. After visually establishing proper signal quality the recording was started. Participants were then interviewed on health, medication, lifestyle and socioeconomic and demographic information after which they filled out a questionnaire on psychological wellbeing. The questionnaire lasted on average 10 min and was completed while quietly sitting in a secluded part of the house/work area. The last 4 min of this quiet sitting period functioned as a baseline. Participants were asked to refrain from exercise during the recording day. The recordings were taken in an unstructured real life setting meaning that no experimental control over the environment was intended or achieved. Twins were not measured on a specific day but with a few exceptions where recordings were rescheduled or collided with holidays, recording was done in the same month (with 46.8% of the pairs acquired within the same week). The median interval between measurements for twin pairs was 9 days, with a range between 0 and 280 days. Of particular relevance to this study, participants were asked to refrain from exercise during the recording day.

**Figure 1:**
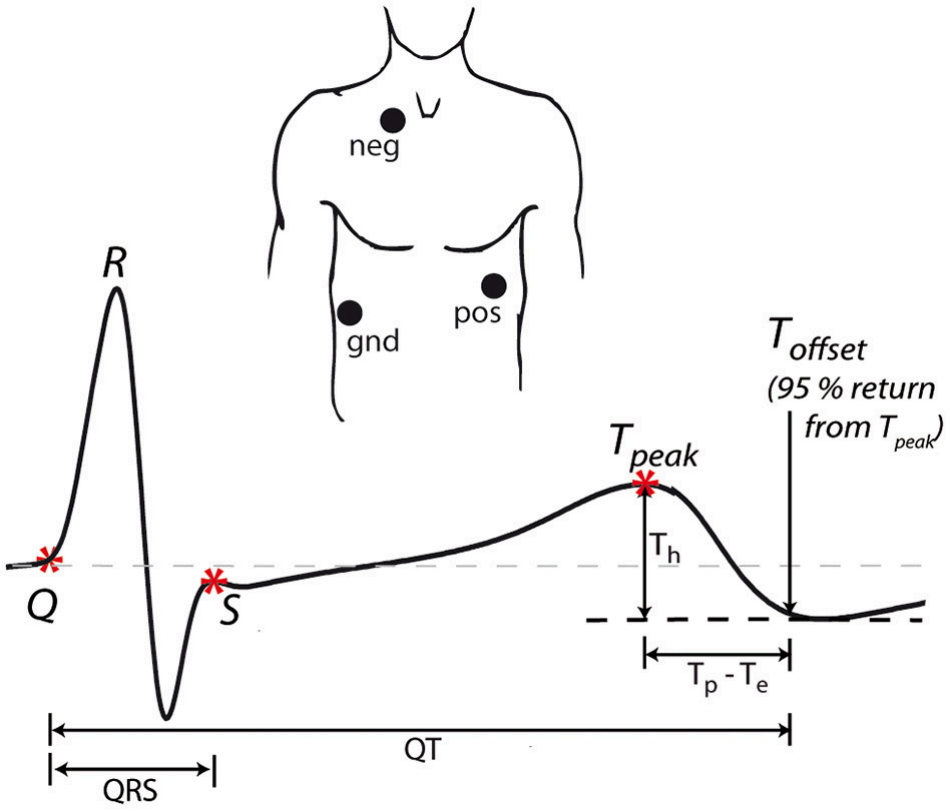
**ECG acquisition**. Three electrodes (positive, negative, and ground) were placed in modified CS5 lead positions to obtain a derived Lead II. The example waveform shows ECG landmarks (^*^) and the measured intervals (QRS, QT, T_p_–T_e_) and amplitudes (T_h_). For all intervals, the time of 95% return from T-peak to the minimum of the T wave was used as the end of repolarization.

Electrodes were placed in modified CS5 lead positions—right subclavicular region 4 cm to the right of the sternum (negative electrode), under the left breast, 4 cm under the nipple (positive electrode) and the lower right thorax (ground electrode)—to obtain a derived Lead II. A typical averaged ECG obtained using the modified CS5 lead positions is shown in Figure 1. The raw ECG signal was imported into the VU-DAMS software (version 3.2, VU University Amsterdam, www.vu-ams.nl) and exported to an ASCII file sampled at 1 kHz for further processing.

In all cases, the T-P interval was used to define the isoelectric line according to standard practice (Goldenberg et al., 2006). Five landmarks were identified on each averaged ECG waveform. (i) Q: first deflection from the isoelectric line after the P-wave, (ii) R: peak of the QRS complex, (iii) S: intersection of S wave upstroke and T-P isoelectric line, (iv) T_peak_: peak of the T-wave, (v) T_offset_: the point 95% of the distance from T-peak to the minimum of the T wave. Since the modified CS5 lead positioning can result in biphasic T-waves (where the end of the T wave overshoots the isoelectric line; Figure 1), the minimum point is typically taken as the end of the T wave (Van Lien et al., 2015). However, since this minimum was sometimes very shallow, the time of the absolute minimum was difficult to precisely identify. Therefore, the time of 95% return from T-peak to the minimum of the T wave was used as the end of repolarization as this could always be accurately determined.

**Figure 2:**
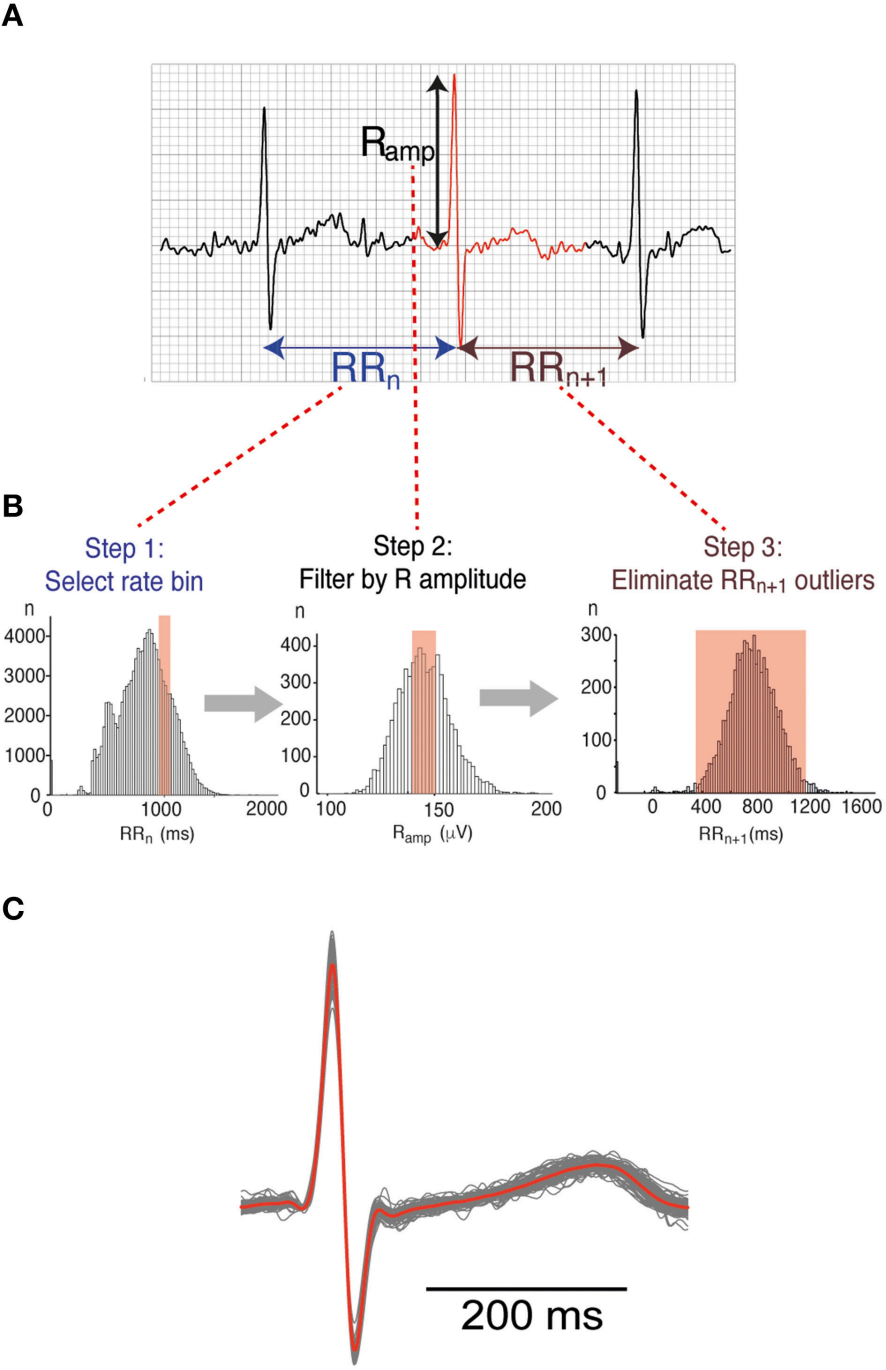
**Beat binning approach**. **(A)** 3 beats from a typical Holter trace. For the beat highlighted in red, three measured parameters are shown: the interval from the peak of the R wave to the previous R peak (RR_n_), the interval from the R peak to the next R peak (RR_n+1_) and the R-amplitude (R_amp_). **(B)** Beats representative of different heart rates were selectively binned according to a 3-step process. The example shown relates to a heart rate bin of 1000 ms RR interval or 60 bpm. Step1: RR_n_ ± 50 ms, Step 2: R_amp_ ± 1 SD, Step 3: RR_n+1_ ± 2SD. **(C)** Family of waveforms extracted according to the binning process illustrated in **(B)**. Individual beats are represented in gray while the averaged waveform is superimposed in red.

From these landmarks we measured the QRS duration (time from Q to S), a measure of ventricular depolarization. We then measured the QT interval (time from Q to T_offset_), T_p_–T_e_ (time from T_peak_ to T_offset_) and T_h_ (amplitude from T_peak_ to T_offset_), all measures of ventricular repolarization (Figure 1). Since P waves could not be consistently measured, due to a combination of signal noise (see for example Figure 2A), lead position and the beat averaging approach, PR interval (a measure of atrial-ventricular conduction) was not included in this study.

### Extraction of averaged ECGs from holter recording

To obtain ECG signals representative of specific heart rates, we used a beat binning approach. R wave peaks were picked using a modified version of R-wave detection software available from the open source PhysioNet resource (www.physionet.org). Every beat of each recording was then binned in a 3-step process based on characteristics of the R wave (Figure 2A) as follows:

**Step 1:** Beats classified by heart rate (RR_n_—The interval from the previous R wave to that of the selected beat) (Figure 2B, Panel 1).
**Step 2:** Each heart rate bin was then filtered by the amplitude of the R wave (R_amp_), to exclude abnormal and/or ectopic beats. Only beats with an R_amp_ within 1 standard deviation (SD) of the mean R_amp_ at that frequency were retained (Figure 2B, Panel 2).
**Step 3:** Beats with an abnormally short or long coupling interval to the subsequent beat (RR_n+1_), defined as > 2SD from the mean, were also excluded (Figure 2B, Panel 3).

Beats within each bin were aligned using the R peak, and the voltage at each timepoint averaged to give an ensemble waveform representative of a particular heart rate (Figure 2C). The relationship between intervals measured from the averaged beat compared to the individual beats in a bin is shown in Supplemental Figure 1. For subsequent analysis of heritability, three heart rates were considered—Low (RR interval 1000 ms/60 bpm), Medium (RR interval 770 ms/78 bpm) and High (RR interval 625/96 bpm). These heart rate bins were chosen purely to give the maximum amount of usable data over as wide a range of heart rates possible. The number of usable averaged waveforms extracted for each heart rate bin for the entire dataset is illustrated in Supplemental Figure 2.

To permit comparison with previous estimates of heritability for ECG parameters, a “resting ECG” was generated for each participant (Supplemental Figure 3). In addition to QRS duration, QT interval, T_p_–T_e_ and T_h_, we also calculated corrected QT intervals for the resting ECGs, using both Bazett's and Fridericia's formulae (Bazett, 1997). Rate corrected QT has consistently been shown to have lower heritability than uncorrected QT (Carter et al., 2000; Haarmark et al., 2011), a trend that was confirmed in our first pass twin correlation calculations (see Supplemental Table 3). Therefore, all further analysis was carried out using uncorrected QT for the resting ECG, as the most stringent comparison to our Holter derived heritability estimates.

All data went through a two-step process of quality control. First, data were deemed unusable if there was an insufficient number of beats at the specified frequency or if there was too much noise. This left 323, 365, and 346 participants for low, medium and high heart rate respectively. Usable data was extracted to derive “resting ECGs” for 427 participants. In stage two, two blinded independent investigators checked the computer-detected points for the Q, R, S, T_peak_, and T_offset_ for every tracing. If the computer-generated data were deemed inaccurate, all measurements for that participant at that particular heart rate were discarded. After quality control, we had data for 322 participants at low heart rate, 357 participants at medium heart rate and 313 participants at high heart rate (this corresponds to 90–99% of traces deemed usable). For the resting ECGs, data from 385 participants were suitable for heritability analysis.

### Genetic analysis based on twin data

Genetic models were fitted to the data using structural equation modeling in the software package Mx (Neale et al., 2003). When data from twins are available, variance in an observed trait is typically decomposed into variance due to latent additive genetic factors (A), non-additive genetic factors (D), common environment (C) shared by family members, and non-shared or unique environment (E). In the classical twin design, which includes monozygotic (MZ) and dizygotic (DZ) twins, estimates of C and D are confounded as the total phenotypic variance, the MZ covariance, and the DZ covariance only provide sufficient information to estimate three out of four parameters (Boomsma et al., 2002). Based on the pattern of twin correlations, we chose to model either an ACE or an ADE model. After establishing the most parsimonious variance components model (ACE or ADE, AE, CE, or E) for each ECG parameter at each frequency two separate analyses were conducted. First, to test if heritability of the resting ECG differs from the Holter ECG and to examine whether the heritability of the different variables extracted from the Holter ECG was rate dependent, quadrivariate genetic models were fitted to the ECG parameters for the three rate groups (1.0, 1.3, and 1.6 Hz) and the resting ECG group (Supplemental Figure 4). A full breakdown of the statistical analysis used to determine which genetic model best as well as to compare equality of heritability estimates is presented in Supplemental Table 5. Second, to examine whether the repolarization parameters were influenced by common or different genetic factors, trivariate genetic models were fitted to the rate specific Holter data (Supplemental Figure 5). From these models we computed the genetic correlations between the three parameters and the contribution of the common genetic factor to the phenotypic correlation as previously described (Neijts et al., 2014). Furthermore, an explicit description of how genetic correlations were calculated in this context is presented in Supplemental Figure 6. The comparison of the fit of restricted models to the full model was performed by means of likelihood-ratio (χ^2^) tests in which the difference in twice the log likelihood (−2LL) between the two models is calculated. When the likelihood-ratio test is significant, the restricted model is considered to fit significantly worse to the data than the fuller model it is tested against. All models regressed the effects of age and sex on the phenotype. A priori, we assumed no quantitative or qualitative sex differences in the variance decomposition to be present so only one MZ and one DZ correlation was estimated for each variable.

## Results

### Rate dependence of ECG characteristics

**Figure 3:**
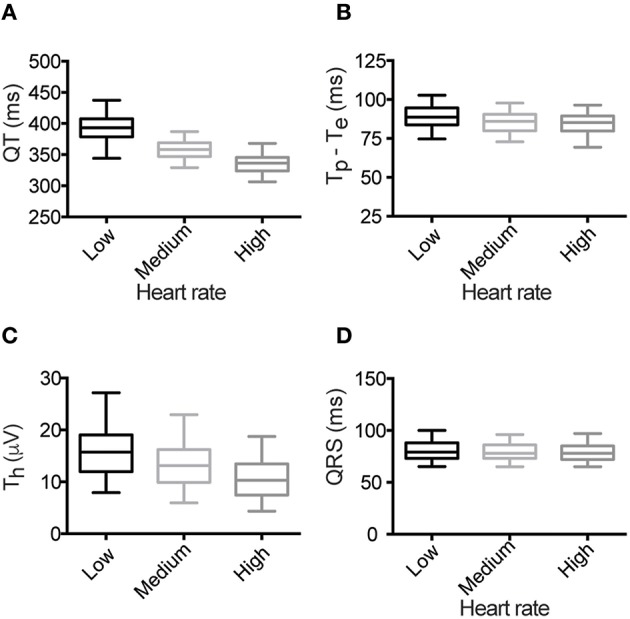
**Heart rate dependence of ECG parameters**. **(A)** QT interval, **(B)** T_p_–T_e_, **(C)** T_h_ and **(D)** QRS duration. For each box plot, the center line marks the mean value, the outer box edges the 25th and 75th percentile and the “whiskers” denote the 5th and 95th percentile.

Measurement of ECG parameters from low, medium and high heart rate averaged beats demonstrated that all measures of repolarization were rate dependent (Figure 3 and Supplemental Table 4). Specifically, both QT interval and T_p_–T_e_ (Figures 3A,B) shortened with faster heart rates, while T_h_ reduced in amplitude (Figure 3C). In contrast, the QRS duration, a measure of depolarization of the myocardium, was not rate dependent Figure 3D).

### Heritability of ECG parameters

**Figure 4:**
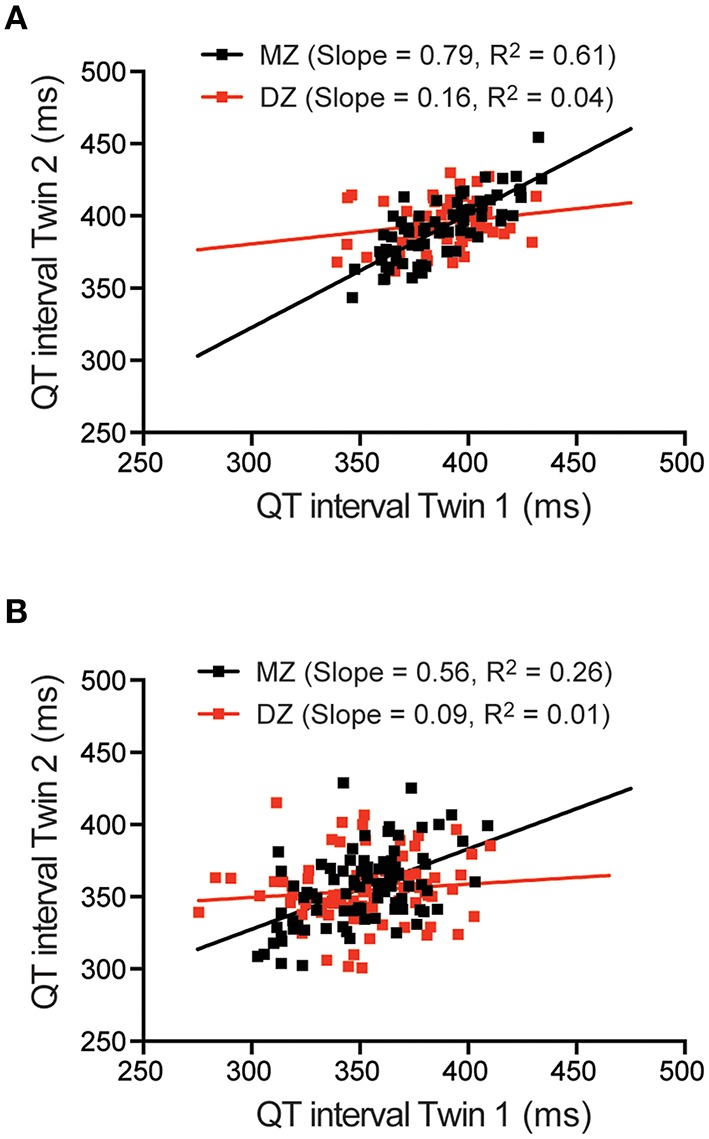
**QT interval correlations in twin pairs**. Scatter plots and linear regression of QT interval for monozygotic (MZ) and dizygotic (DZ) twin pairs measured from low rate Holter ECG **(A)** or resting ECG **(B)**.

Scatterplots of the MZ vs. DZ pairwise correlations for QT intervals obtained from the low heart rate ECGs (Figure 4A) and from the resting ECG (Figure 4B) are shown in Figure 4. From these plots it is clear that there is a greater correlation in the monozygotic (MZ) twins compared to the dizygotic (DZ) twins, consistent with there being a significant genetic contribution to QT interval. The twin correlations for each of the 4 ECG parameters at different heart rates are summarized in Table 1. MZ pair correlations were higher than the DZ correlation in all cases indicating that there is a genetic component for all parameters.

**Table 1:** **Monozygotic and dizygotic twin correlations from the saturated model**.

**ECG parameter**	**Rate**	**MZ correlation**	**DZ correlation**
TpTe	Low	0.62 (0.36–0.77)	0.12 (−0.27–0.46)
	Medium	0.69 (0.49–0.81)	0.07 (−0.40–0.49)
	High	0.55 (0.23–0.73)	−0.39 (−0.71–0.34)
	Resting	0.55 (0.29–0.71)	0.00 (−0.35–0.36)
Th	Low	0.65 (0.34–0.80)	0.34 (−0.05–0.61)
	Medium	0.66 (0.43–0.79)	0.46 (0.10–0.69)
	High	0.53 (0.22–0.72)	0.42 (0.02–0.67)
	Resting	0.54 (0.26–0.71)	0.06 (−0.25–0.36)
QT	Low	0.71 (0.49–0.82)	0.07 (−0.33–0.45)
	Medium	0.62 (0.38–0.76)	0.15 (−0.20–0.50)
	High	0.50 (0.18–0.69)	0.25 (−0.17–0.57)
	Resting	0.51 (0.23–0.69)	0.14 (−0.16–0.41)
QRS	Low	0.52 (0.19–0.72)	−0.10 (−0.46–0.30)
	Medium	0.52 (0.23–0.71)	−0.10 (−0.43–0.27)
	High	0.42 (0.12–0.62)	0.07 (−0.47–0.55)
	Resting	0.34 (0.06–0.56)	−0.14 (−0.44–0.21)

99% CIs are shown in parentheses.

Having established that there is a genetic component to each of the ECG parameters, we performed formal twin modeling to estimate heritability. Univariate analysis showed that all estimation models could be reduced to combinations of additive genetic and unique environmental factors, or an AE model without significant loss of fit.

A quadrivariate AE model including low, medium and high rate Holters and the resting ECG (see Supplemental Figure 4) was fitted to the data to estimate heritability. On the whole, repolarization parameters (QT, T_p_–T_e_, and T_h_) showed a larger heritability estimate (ranging from 56 to 72% at low heart rates) than QRS, the depolarization parameter (41% at low heart rate) (Table 2). Significantly higher heritability estimates could be obtained when measured from Holter data compared to the resting ECG for QT interval (*p* < 0.01) and T_h_ (*P* < 0.05). Likewise, significant rate dependence of the heritability estimate from Holter recordings was observed for QT interval (*p* < 0.01) and T_h_ (*P* < 0.05). A formal statistical analysis of these relationships is presented in Supplemental Table 5.

**Table 2:** **Heritability of ECG parameters for Low, Medium, and High heart rate Holter and resting ECG**.

**Parameter**	**Best Model**	**ECG type**	**Heritability (99% CI)**
TpTe	AE	Low	56% (32–73)
		Medium	63% (36–79)
		High	52% (21–73)
		Resting	56% (30–72)
Th	AE	Low	72% (53–83)
		Medium	68% (49–80)
		High	58% (36–73)^*^
		Resting	55% (31–71)^*^
QT	AE	Low	69% (48–82)
		Medium	58% (33–75)
		High	34% (14–53)^**^
		Resting	40% (18–58)^**^
QRS	AE	Low	41% (10–64)
		Medium	41% (13–63)
		High	42% (14–62)
		Resting	32% (7–55)

Asterisks denote statistical difference compared to low rate Holter where

** p < 0.01 and

* p < 0.05.

Full statistical analysis is presented in Supplemental Table 5.

### Rate dependence of genetic factors influencing holter ECG parameters

To further investigate the rate dependence observed above, we tested the extent of the correlation among ECG parameters measured at different heart rates and to what extent genetic or environmental factors explained this phenotypic correlation (see Table 3). Phenotypic correlations across heart rates ranged from 0.47 to 0.94 and were highest for T_h_ and QRS. The genetic contributions to these phenotypic correlations were higher for the repolarization parameters than for QRS (the depolarization parameter). The 3rd column of Table 3 lists the genetic correlation between rates for individual ECG parameters. With the exception of the comparisons between T_p_ and T_e_ at low and high heart rates and QT at low and high heart rates, all the other genetic correlations are very high, up to 1.0 for QRS between medium and high heart rates. This indicates that largely the same genetic factors influence the different ECG parameters irrespective of the heart rate at which they are being measured.

**Table 3:** **Phenotypic and genetic correlation between different heart rates for individual ECG parameters**.

**Parameter/Frequency comparison**	**Phenotypic correlation**	**Genetic correlation between rates**	**Contribution of genetic factors to phenotypic co-variance**
**Tp-Te**			
Low-medium	0.76 (0.67–0.83)	0.88 (0.73–0.97)	68% (39–86%)
Low-high	0.56 (0.41–0.67)	0.56 (0.20–0.80)	54% (14–82%)
Medium-high	0.76 (0.68–0.83)	0.89 (0.72–0.97)	66% (32–87%)
**Th**			
Low-medium	0.93 (0.90–0.95)	0.98 (0.94–1.00)	74% (55–85%)
Low-high	0.82 (0.75–0.87)	0.91 (0.79–1.00)	72% (51–85%)
Medium-high	0.93 (0.90–0.95)	0.97 (0.93–1.00)	66% (45–79%)
**QT**			
Low-medium	0.70 (0.61–0.78)	0.77 (0.57–0.92)	69% (42–86%)
Low-high	0.47 (0.32–0.59)	0.52 (0.15–0.78)	54% (14–81%)
Medium-high	0.72 (0.64–0.79)	0.84 (0.62–0.95)	52% (24–72%)
**QRS**			
Low-medium	0.94 (0.92–0.96)	0.97 (0.84–1.00)	42% (12–65%)
Low-high	0.86 (0.81–0.90)	0.95 (0.79–1.00)	46% (13–69%)
Medium-high	0.90 (0.87–0.93)	1.00 (0.95–1.00)	46% (15–68%)

99% CIs are shown in parentheses.

### Genetic correlations between the repolarization parameters

We next investigated whether the repolarization parameters (QT, T_h_, and T_*p*_–T_e_) were influenced by similar or different genetic factors. At low and medium heart rates, phenotypic correlation between the repolarization parameters were significant, but of modest size, and the phenotypic covariance observed was almost entirely caused by genetic factors (Table 4, last column). Despite this, the genetic correlation between parameters was low. For example, at low heart rates, genetic correlations were 0.38 for QT/T_p_–T_e_, −0.42 for QT/T_h_ and −0.58 for T_p_–T_e_/T_h_. At high heart rates, there were no significant phenotypic or genetic correlations between repolarization parameters.

**Table 4:** **Phenotypic and genetic correlation between repolarization ECG parameters at each heart rate**.

**Frequency/Parameter comparison**	**Phenotypic correlation**	**Genetic correlation between parameters**	**Contribution of genetic factors to phenotypic co-variance**
**LOW**			
QT-TpTe	0.38 (0.22–0.51)	0.38 (0.05–0.63)	65% (9–97%)
QT-Th	−0.28 (−0.43 to −0.12)	−0.42 (−0.70 to −0.12)	100% (39–00%)
TpTe-Th	−0.39 (−0.52 to −0.24)	−0.58 (−0.93 to −0.26)	92% (47–100%)
**MEDIUM**			
QT-TpTe	0.32 (0.16–0.45)	ns	ns
QT-Th	−0.25 (−0.40 to −0.09)	−0.46 (−0.82 to −0.15)	100% (58–100%)
TpTe-Th	−0.18 (−0.33 to −0.02)	−0.36 (−0.75 to −0.06)	100% (58–100%)
**HIGH**			
QT-TpTe	0.31 (0.15–0.46)	ns	ns
QT-Th	ns	ns	–
TpTe-Th	ns	ns	–

99% CIs are shown in parentheses.

## Discussion

In this study we present the first measurement of the heritability of repolarization parameters from Holter ECGs. To achieve this we used an extended selective beat binning approach to extract data from Holter recordings that is representative of specific heart rates. Twin modeling based on this approach resulted in higher estimates of heritability for T_h_ and QT interval compared to the resting ECG. Furthermore, our approach allowed us to interrogate the overlap between the genetic factors influencing individual ECG parameters at different heart rates as well as between the genetic factors that determine the different repolarization parameters at each heart rate separately. Our data therefore demonstrates the potential for analysis of Holter ECGs to give a more complete insight into the genetic underpinnings of the cardiac electrical system compares to what can be obtained from the resting ECG.

### Beat binning approach

The beat binning approach used here, binned by heart rate followed by an efficient removal of outliers based on R-amplitude and the RR interval of the subsequent beat, permits extraction of low noise, averaged beats, representative of different heart rates. Not only does this approach allow for genuine like-for-like analysis between participants, i.e., we can directly compare a 60 bpm waveform from each subject, it also allows us to perform analysis of rate dependent trends in phenotype and heritability that is not possible considering the resting ECG alone. Another approach to examining rate dependent trends in ambulatory Holter ECG data would be to use beat-to-beat analysis. However, beat-to-beat analysis is complicated by significant inter-beat variability and signal noise. Rather than analysing the data on such a per-beat basis, our approach selectively classifies beats, based on unambiguous properties of the R-wave, meaning the complexities of beat-to-beat analysis are avoided. Furthermore, most Holter analysis packages in clinical use routinely annotate R-waves (e.g., GE Mars, Spacelabs, Philips) while many open source databases of Holter data are preannotated (e.g., THEW, Physionet). As a result the preprocessing of signals required for binning of data is already available in most cases. By way of validation of our beat binning approach, analysis of rate dependent trends in the characteristics of the idealized waveforms were consistent with previously published data. Specifically, QT interval and T_p_–T_e_ shortened, and T_h_ amplitude decreased with faster rates (Lehmann and Yang, 2001; Batchvarov et al., 2002; Couderc et al., 2007; Malik et al., 2008). Conversely, the QRS interval was rate-independent in our data, also consistent with previous observations (Simoons and Hugenholtz, 1975).

It should be noted that any averaging process represents a compromise between getting a more representative overall response vs. losing sensitivity to detect more subtle changes that can occur on a beat-to-beat basis, such as QT hysteresis effects and differences in autonomic tone between night and day. For example, the potential confounding effect of QT hysteresis, is that multiple QT intervals may be binned together with a single RR interval after rapid changes in heart rate, when the QT interval change is delayed relative to the heart rate acceleration or deceleration (Malik, 2005). One way to tackle this is to bin beats according to stability of the RR interval over the previous 30 beats for example rather than just the immediately preceding beat (Badilini et al., 1999; Malik, 2005). Unfortunately, unless the participant is kept in very controlled conditions (as might be possible in the context of a clinical trial), the dataset is decimated by this approach, and much of the Holter record needs to be eliminated. In our case, where Holter recordings were acquired in a true unstructured real life setting, with no experimental control over the environment, this was certainly the case. We analyzed our data to quantify the number of useable individual beats in each record based on the RR interval stability of a period of 30 beats preceding the index beat. The criteria applied were that the index beat was excluded from the binning process if any of the preceding 30 beats fell outside of a threshold level of variability from the mean RR interval of that 30 beat window. The thresholds considered were ±5 and ±10%. This corresponds, for example, to RR interval windows of 100 and 200 ms at 60 bpm. This approach excluded 91.8 ±7% and 61 ± 16% of our data (SD; *n* = 163) at the variability thresholds of 5 and 10% respectively. We were therefore unable to apply this additional level of selection to our binning approach. While we acknowledge this limitation, this was a necessary compromise to preserve the dataset such that we had the statistical power to tackle the main aims of our project—i.e., to improve heritability measures of ECG parameters related to repolarization and to demonstrate rate dependence to these heritabilities. We achieved both of these aims despite these potentially confounding factors, and suggest that in the context of a “perfect” dataset, the reported differences might be even greater than we are able to resolve.

### Heritability of ECG parameters

Heritability of parameters related to depolarization (QRS duration) was lower than for repolarization-related parameters (QT interval, T_p_–T_e_, and T_h_), with a maximum heritability of 42% for QRS duration, compared to between 63 and 72% for repolarization parameters. Previous studies have not been able to demonstrate a significant contribution of additive genetic factors to QRS duration, possibly related to sample size (Mathers et al., 1961; Havlik et al., 1980; Russell et al., 1998; Mutikainen et al., 2009). Our study is therefore the first to present a formal measure of the role of additive genetics in determination of this parameter.

For parameters related to repolarization, heritability measured from our rate specific Holter data was at least comparable, or higher, than our measures based on resting ECGs. In particular, significantly higher heritabilities of QT (69% for low rate Holter compared to 40% for the resting ECG, *P* < 0.01) and T_h_ (72% from low rate Holter compared to 55% for the resting ECG, *P* < 0.05) were measured from the Holter data. The same trend (of increased heritability measured from the Holter ECG) was also evident when our estimates were compared to previously published data based on resting ECGs. For example, the most comprehensive twin study of repolarization parameters in resting ECGs to date reported heritability of 67% for QT interval, 46% for T_p_–T_e_ and between 34 and 47% for T_h_, after correction for confounding factors such as age and sex (Haarmark et al., 2011), in comparison to our measures of 69, 63, and 72% respectively. More broadly, other studies have reported genetic contributions of zero (Mutikainen et al., 2009), 25% (Carter et al., 2000), 36% (Russell et al., 1998), and 60% (Dalageorgou et al., 2008) to QT interval, and between zero and 72% for T_h_ (Haarmark et al., 2011). The estimates for T_h_ however, are heavily dependent on the lead selected from the resting ECG. Of most direct comparison to our data, Mutikainen reported heritability of 61% when measured from lead II (Mutikainen et al., 2009). These results show that by considering data extracted from Holter ECGs, higher heritability can be measured for most ECG parameters and that rate specific ECG waveforms extracted from Holter recordings give a more precise measure of the effect of the underlying genotype than the resting ECG. It should be noted however, that particularly at low rates, the binning approach used in this study does not discriminate between day and night periods meaning nocturnal intervals associated with low autonomic tone will be mixed with diurnal intervals at the same heart rate. It is therefore likely that our heritability estimates are still underestimates of the true contribution of additive genetics to ECG parameters at lower heart rates.

### Rate dependence of genetic factors influencing the ECG

A major advantage of our approach to analysing Holter data is that we are able to measure the rate dependence of genetic factors influencing the ECG—something that is not tractable using the resting ECG. This is an important consideration in understanding the rate dependent changes that occur in T wave morphology in both physiological (Smetana et al., 2003; Sadrieh et al., 2013) and pathophysiological states (Couderc et al., 2007). Our data showed that for QT interval and T_h_, heritability was dependent on the heart rate. Possible explanations for these observations included non-genetically determined adrenergic responses, such as the conditioning effect of exercise. In contrast, no significant effect of heart rate could be determined for QRS and T_p_–T_e_.

Additional analysis showed that the genetic contribution to phenotypic covariance between rates varied for different parameters (but was higher for all repolarization parameters compared to QRS duration). Furthermore, this genetic contribution was in general greatest at low heart rates, while at faster heart rates, environmental factors played an increasing role in defining ECG characteristics (Table 3, column 4). In considering the genetic correlation between rates, our analysis showed that the overall genetic factors that influenced each of the ECG parameters between heart rates largely overlapped, as indicated by the very high genetic correlation between rates for individual parameters (see Table 3 column 3). These data therefore support the concept that the same components of the rhythmonome define individual ECG parameters regardless of the heart rate.

### Genetic overlap between repolarization parameters

In addition to examining the genetic overlap between different heart rates for individual ECG parameters, we also examined the genetic overlap between pairs of repolarization parameters at a given heart rate. This latter analysis demonstrated that even though the genetic contribution to phenotypic covariance between repolarization parameters is very high, the genetic correlation between parameters is relatively low (between 0.38 for QT and T_p_–T_e_ and 0.58 for T_p_–T_e_ and T_h_ at low heart rates) suggesting there is relatively little overlap between the genetic factors that define these parameters. This is consistent with our previous studies that showed that individual ECG parameters had very different sensitivities to variability in cardiac ion channel genes (Sadrieh et al., 2013, 2014). This is an important observation as it establishes that in order to fully describe the effect of an individual's genotype, multiple parameters describing the ECG waveform must be measured.

### Clinical perspective

The QT interval is a commonly used diagnostic parameter that reflects the duration of cardiac repolarization. In the clinical setting, either shortening or prolongation of QT has long been associated with risk of sudden death, while drugs that prolong the QT interval increase risk of ventricular arrhythmias. More recently, characterization of T-wave morphology has been explored in characterizing repolarization, improving the discrimination between subtypes of LQTS. Our work expands on this by showing that T_p_-T_e_ and T_h_, as well as QT, have significant heritable components and more importantly that the genetic basis of this heritability differs for the different repolarization parameters. A consequence of this observation is that one must measure multiple ECG parameters from a patient to fully reflect the individual's genotype, since measuring one individual parameter, the QT interval for example, only represents a fraction of the whole cardiac ion channel gene complement.

### Limitations

The biphasic T wave produced by the electrode configuration in this study precluded measurement of the end of the T wave using the intersection of a tangent to the downslope of the T wave with the isoelectric line. Instead, T_offset_ was used as the end of the T wave in this study as previously published (Van Lien et al., 2015) and described here (see Materials and Methods). The major limitation of our approach is that in binning beats based on RR interval we are unable to take into account effects related to changes in autonomic tone, circadian variability in QT interval or QT hysteresis. However, this was a necessary compromise imposed by the need to maintain the volume of data for individual heart rates that gives us the statistical power to measure rate dependent changes in heritability for example. We would suggest the differences we report might be greater in the context of a “perfect” dataset.

## Conclusion

In conclusion, our novel beat binning approach to analysis of the Holter ECG has allowed the first rate specific estimate of the heritability of ECG parameters from the Holter ECG. Our data demonstrates that higher measures of heritability can be estimated from the Holter than the resting ECG, suggesting that this approach allows a more complete assessment of the genetic contribution to cardiac electrical activity. Furthermore, we show rate dependence in the genetic—environmental determinants of the ECG that has not previously been tractable. Future potential uses of this type of analysis include deeper dissection of the ECG of participants with inherited cardiac electrical disease.

## Author contributions

EH, MN, and AS contributed equally as co-first authors. AH and ED contributed equally as senior authors. AH, ED, EH, JV conceived the study and analyzed and interpreted data. MN, DB, GW, ED acquired data. AS, MB, MI, RS, CH analyzed and interpreted data. All authors drafted the manuscript, approved this version and are accountable for the work.

## Funding

This work was supported by grants from the National Health and Medical Research Council of Australia (#1006016) and the St. Vincent's Clinic Foundation. AH is supported by an Australian Research Council Future Fellowship (FT110100075) and JV is supported by an NHMRC Senior Research Fellowship (#1019693).

### Conflict of interest statement

The authors declare that the research was conducted in the absence of any commercial or financial relationships that could be construed as a potential conflict of interest.

## Abbreviations

ECG: Electrocardiogram
MZ: Monozygotic
DZ: Dizygotic
SCD: Sudden Cardiac Death
LQTS: Long QT Syndrome.
