# Supplementary material for: Heritability of ECG Biomarkers in the Netherlands Twin Registry Measured from Holter ECGs
Source: Front Physiol. 2016 Apr 29;7:154. doi: 10.3389/fphys.2016.00154 (PMC4850154; doi:10.3389/fphys.2016.00154)
Supplement: Supplementary file 1 [file Table1.PDF]

**Medication (Anatomical therapeutic chemical classification code)**	**Number of participants**
Anti Hypertensives (ATC C02)	**1**
Diuretics (ATC C03)	**8**
Beta Blockers (ATC C07)	**7**
Ca Channel blockers (ATC C08)	**3**
Renin-Angiotension system agents (ATC C09)	**10**
Lipid modifiers (ATC C10)	**7**
Anti Psoriatics (ATC D05)	**2**
Hormone replacement therapy (ATC G03)	**3**
Urologicals (ATC G04)	**2**
Thryoid therapy (ATC H03)	**6**
Endocrine therapy (ATC L02)	**1**
Immunosupressants (ATC L04)	**3**
Immunostimulants (ATC L03)	**1**
Anti Epileptics (ATC N03)	**5**
Anti Parkinson drugs (ATC N04)	**1**
Psycholeptics (ATC N05)	**15**
Psychoanaleptics (ATC N06)	**18**
Obstructive airways disease agents (ATC R03)	**17**

Supplemental Table 1: Participants taking medications with potential to alter ECG intervals. Anatomical Therapeutic Chemical (ATC) Classification Codes are shown.
