# Supplementary material for: Heritability of ECG Biomarkers in the Netherlands Twin Registry Measured from Holter ECGs
Source: Front Physiol. 2016 Apr 29;7:154. doi: 10.3389/fphys.2016.00154 (PMC4850154; doi:10.3389/fphys.2016.00154)
Supplement: Supplementary file 2 [file Table2.PDF]

ECG parameter	Rate	MZ correlation	DZ correlation
TpTe	Low	0.58 (0.35-0.72)	0.01 (-0.32-0.33)
	Medium	0.67 (0.51-0.78)	0.11 (-0.27-0.44)
	High	0.63 (0.44-0.75)	-0.18 (-0.57-0.36)
	Resting	0.56 (0.36-0.70)	0.08 (-0.25-0.37)
Th	Low	0.58 (0.34-0.74)	0.38 (0.08-0.59)
	Medium	0.56 (0.33-0.71)	0.43 (0.15-0.62)
	High	0.54 (0.29-0.71)	0.39 (0.11-0.61)
	Resting	0.45 (0.19-0.63)	0.19 (-0.06-0.41)
QT	Low	0.69 (0.52-0.80)	0.07 (-0.30-0.41)
	Medium	0.57 (0.37-0.71)	0.24 (-0.07-0.50)
	High	0.47 (0.20-0.65)	0.25 (-0.09-0.52)
	Resting	0.52 (0.29-0.68)	0.09 (-0.17-0.34)
QRS	Low	0.46 (0.20-0.64)	0.10 (-0.24-0.41)
	Medium	0.49 (0.25-0.66)	0.08 (-0.25-0.39)
	High	0.39 (0.14-0.59)	0.25 (-0.24-0.58)
	Resting	0.30 (0.07-0.50)	0.00 (-0.29-0.29)

Supplemental Table 2: Monozygotic and dizygotic twin correlations from the saturated model including participants taking cardioactive medication. 99% CIs are shown in parentheses
