# Supplementary material for: Heritability of ECG Biomarkers in the Netherlands Twin Registry Measured from Holter ECGs
Source: Front Physiol. 2016 Apr 29;7:154. doi: 10.3389/fphys.2016.00154 (PMC4850154; doi:10.3389/fphys.2016.00154)
Supplement: Supplementary file 3 [file Table3.PDF]

	MZ	DZ
QT	.52 (95)	.11 (76)
QT_{cb}	.34 (95)	.10 (76)
QT_{cf}	.34 (95)	.05 (76)

Supplemental Table 3: Monozygotic and dizygotic twin correlations for uncorrected QT, Bazett corrected QT (QT_{cb}) and Frideracia corrected QT (QT_{cf}). Numbers of twin pairs are shown in parentheses.
