# Supplementary material for: Heritability of ECG Biomarkers in the Netherlands Twin Registry Measured from Holter ECGs
Source: Front Physiol. 2016 Apr 29;7:154. doi: 10.3389/fphys.2016.00154 (PMC4850154; doi:10.3389/fphys.2016.00154)
Supplement: Supplementary file 4 [file Table4.PDF]

Heart rate	T_p-T_e (ms)	T_h (mV)	QT (ms)	QRS (ms)
**Low (60bpm)**	88.9 ± 8.1	1.6 ± 0.6	392.9 ± 20.4	80.41 ± 10.6
**Medium (78bpm)**	85.2 ± 8.2	1.4 ± 0.5	357.9 ± 17.3	79.20 ± 10.0
**High (96bpm)**	84.2 ± 8.6	1.1 ± 0.5	335.9 ± 18.4	79.3 ± 10.3
**Resting**	79.0 ± 10.5	1.5 ± 0.7	354.1 ± 25.2	78.8 ± 9.3

Supplemental Table 4: Rate dependent analysis of ECG parameters. Mean data ± SD are shown.
