# Supplementary material for: Heritability of ECG Biomarkers in the Netherlands Twin Registry Measured from Holter ECGs
Source: Front Physiol. 2016 Apr 29;7:154. doi: 10.3389/fphys.2016.00154 (PMC4850154; doi:10.3389/fphys.2016.00154)
Supplement: Supplementary file 5 [file Table5.PDF]

Supplemental Table 5: Statistical analysis of the quadrivariate model

Parameter	Model	df	Model	AIC	-2LL	vs	$\Delta\chi^2$	Δ df	p	Frequency	A	E
Tp_Te	1	1104	AE	90.320	2298.320					Low	0.56 (0.32-0.73)	0.44 (0.27-0.68)
										Medium	0.63 (0.36-0.79)	0.37 (0.21-0.64)
										High	0.52 (0.21-0.73)	0.48 (0.27-0.79)
										Resting	0.56 (0.30-0.72)	0.44 (0.28-0.70)
	**2**	**1107**	**low=medium=high=resting**	**86.672**	**2300.672**	**1**	**2.352**	**3**	**.503**			
	**3**	**1105**	**low=resting**	**88.320**	**2298.320**	**1**	**0**	**1**	**.995**			
	**4**	**1105**	**medium=resting**	**89.000**	**2299.000**	**1**	**0.681**	**1**	**.409**			
	**5**	**1105**	**high=resting**	**88.415**	**2298.415**	**1**	**0.096**	**1**	**.757**			
	**6**	**1106**	**low=medium=high**	**88.648**	**2300.648**	**1**	**2.329**	**2**	**.312**			
	**7**	**1105**	**low=high**	**88.419**	**2298.419**	**1**	**0.1000**	**1**	**.752**			
	**8**	**1105**	**medium=high**	**90.073**	**2300.073**	**1**	**1.754**	**1**	**.185**			
QT	1	1104	AE	2038.801	4246.801					Low	0.69 (0.48-0.82)	0.31 (0.18-0.52)
										Medium	0.58 (0.33-0.75)	0.42 (0.25-0.67)
										High	0.34 (0.14-0.53)	0.66 (0.47-0.86)
										Resting	0.40 (0.18-0.58)	0.60 (0.42-0.82)
	2	1107	low=medium=high=resting	2051.523	4265.523	1	18.722	3	.000			
	3	1105	low=resting	2047.349	4257.349	1	10.548	1	.001			
	**4**	**1105**	**medium=resting**	**2041.619**	**4251.619**	**1**	**4.819**	**1**	**.028**			
	**5**	**1105**	**high=resting**	**2037.257**	**4247.257**	**1**	**0.456**	**1**	**.499**			
	6	1106	low=medium=high	2050.834	4262.834	1	16.033	2	.000			
	7	1105	low=high	2049.598	4259.598	1	12.797	1	.000			
	8	1105	medium=high	2045.416	4255.416	1	8.616	1	.003			
Th	1	1104	AE	-1753.587	454.413					Low	0.72 (0.53-0.83)	0.28 (0.17-0.47)
										Medium	0.68 (0.49-0.80)	0.32 (0.20-0.51)
										High	0.58 (0.36-0.73)	0.42 (0.27-0.64)
										Resting	0.55 (0.31-0.71)	0.45 (0.29-0.69)
	**2**	**1107**	**low=medium=high=resting**	**-1751.869**	**462.131**	**1**	**7.717**	**3**	**.052**			
	**3**	**1105**	**low=resting**	**-1750.011**	**459.989**	**1**	**5.575**	**1**	**.018**			
	**4**	**1105**	**medium=resting**	**-1751.779**	**458.221**	**1**	**3.807**	**1**	**.051**			
	**5**	**1105**	**high=resting**	**-1755.411**	**454.589**	**1**	**0.176**	**1**	**.675**			
	**6**	**1106**	**low=medium=high**	**-1752.387**	**459.613**	**1**	**5.200**	**2**	**.074**			
	**7**	**1105**	**low=high**	**-1751.453**	**458.547**	**1**	**4.134**	**1**	**.042**			
	**8**	**1105**	**medium=high**	**-1750.799**	**459.201**	**1**	**4.788**	**1**	**.029**			

Continued overleaf

QRS	1	1104	AE	-599.646	1608.354					Low	0.41 (0.10-0.64)	0.59 (0.36-0.90)
										Medium	0.41 (0.13-0.63)	0.59 (0.37-0.87)
										High	0.42 (0.14-0.62)	0.58 (0.38-0.86)
										Resting	0.32 (0.07-0.55)	0.68 (0.45-0.93)
	**2**	**1107**	**low=medium=high=resting**	**-603.382**	**1610.618**	**1**	**2.263**	**3**	**.520**			
	**3**	**1105**	**low=resting**	**-600.656**	**1609.344**	**1**	**0.990**	**1**	**.320**			
	**4**	**1105**	**medium=resting**	**-599.579**	**1610.421**	**1**	**2.067**	**1**	**.150**			
	**5**	**1105**	**high=resting**	**-599.832**	**1610.168**	**1**	**1.813**	**1**	**.178**			
	**6**	**1106**	**low=medium=high**	**-603.624**	**1608.376**	**1**	**.022**	**2**	**.989**			
	**7**	**1105**	**low=high**	**-601.624**	**1608.376**	**1**	**.022**	**1**	**.883**			
	**8**	**1105**	**medium=high**	**-601.641**	**1608.359**	**1**	**.004**	**1**	**.947**			

df = degrees of freedom; Model = specification of the model that is tested; AIC = Akaike's Information Criterion; -2LL = minus twice the logarithm of the likelihood; vs = the model against which this submodel is tested; $\Delta\chi^2$ = model fit statistic: difference in -2LL of two nested models; Δdf = difference in the number of parameters between the two models; p = p-value; A and E = proportions of variance explained by additive and unique environmental effects for the most parsimonious AE model.
