# Supplementary material for: Heritability of ECG Biomarkers in the Netherlands Twin Registry Measured from Holter ECGs
Source: Front Physiol. 2016 Apr 29;7:154. doi: 10.3389/fphys.2016.00154 (PMC4850154; doi:10.3389/fphys.2016.00154)
Supplement: Supplementary file 6 [file Image1.PDF]

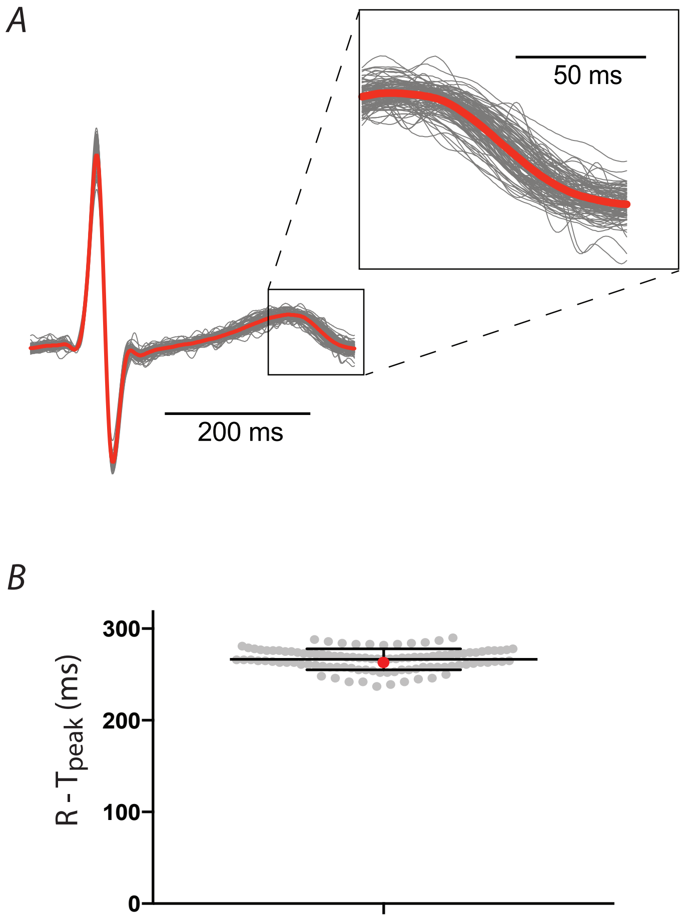

Supplemental Figure 1: Relationship between measured intervals from invididual versus averaged waveforms. A) Family of waveforms extracted at 60 bpm using the multiparameter selective binning approach described in Figure 2. Individual beats are represented as grey lines, while the averaged beat for this family is superimposed in red. The inset shows a zoomed view of the descending limb of the T wave.

B) Summary of R-Tpeak intervals measured from panel (A). Intervals measured from individual beats are shown in grey, mean ± SD (266.5 ± 11 ms, n=110) of the individual population is shown in black, interval measured from the averaged beat (264 ms) is shown in red.
