# Supplementary material for: Heritability of ECG Biomarkers in the Netherlands Twin Registry Measured from Holter ECGs
Source: Front Physiol. 2016 Apr 29;7:154. doi: 10.3389/fphys.2016.00154 (PMC4850154; doi:10.3389/fphys.2016.00154)
Supplement: Supplementary file 7 [file Image2.PDF]

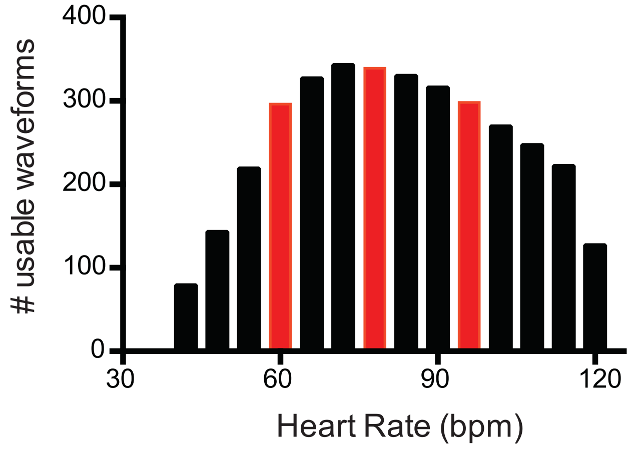

Supplemental Figure 2: Number of usable averaged waveforms at different heart rates. The three heart rate bins used in this study representing low heart rate (60 bpm), medium heart rate (78 bpm) and high heart rate (96 bpm) are highlighted red. These heart rate bins were chosen to give as wide a range of heart rates possible, with the maxium amount of usable data.
