# Supplementary material for: Heritability of ECG Biomarkers in the Netherlands Twin Registry Measured from Holter ECGs
Source: Front Physiol. 2016 Apr 29;7:154. doi: 10.3389/fphys.2016.00154 (PMC4850154; doi:10.3389/fphys.2016.00154)
Supplement: Supplementary file 8 [file Image3.PDF]

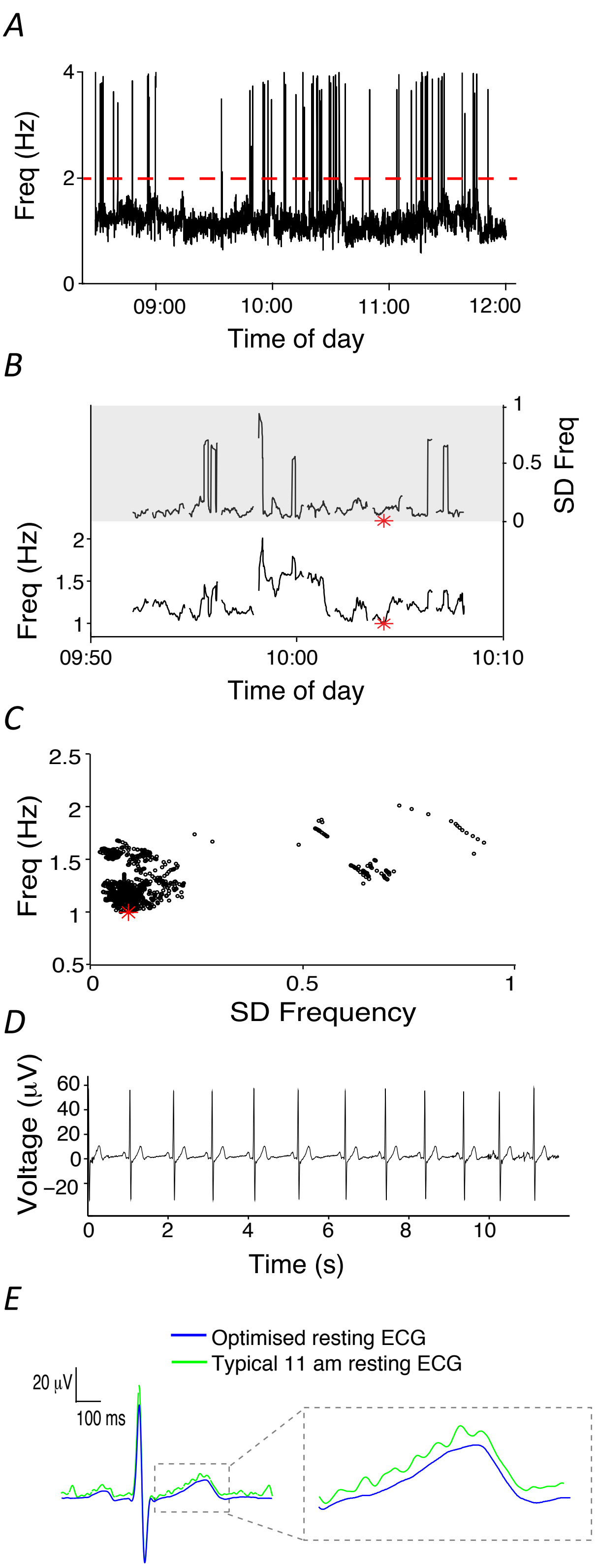

Supplemental Figure 3: Extraction of resting ECG. A) R peak frequency in the period between 8am and12pm for a typical participant. Beats with a frequency above 2Hz (120bpm, red dashed line) were considered non-physiological and excluded from further analysis. Within the entire patient dataset, the region between 9.50am and 10.10am showed the lowest SD of RR interval on average. This region was therefore selected for extraction of a typical 'resting ECG' from each participant. B-C) The 10s with the lowest frequency and standard deviation (red asterisk) between 9:50am and 10:10am was selected for each participant, ensuring we extracted the 'best' resting ECG in terms of stability and resting heart rate (average heart rate was 67 ± 0.6 bpm or 1.12 ± 0.01 Hz (SEM; n=385)). D) 10s of Holter ECG activity selected according to the criteria identified in B-C. E) An averaged beat from the 10s of data presented in D (optimised resting ECG, Blue) compared to an averaged beat taken at random from 10s of data at 11am.
