# Supplementary figures and images for: Heritability of ECG Biomarkers in the Netherlands Twin Registry Measured from Holter ECGs
Source: Front Physiol. 2016 Apr 29;7:154. doi: 10.3389/fphys.2016.00154 (PMC4850154; doi:10.3389/fphys.2016.00154)

**Supplemental Figure 4: Quadrivariate model**

$r_{MZ} = 1, r_{DZ} = 0.5$

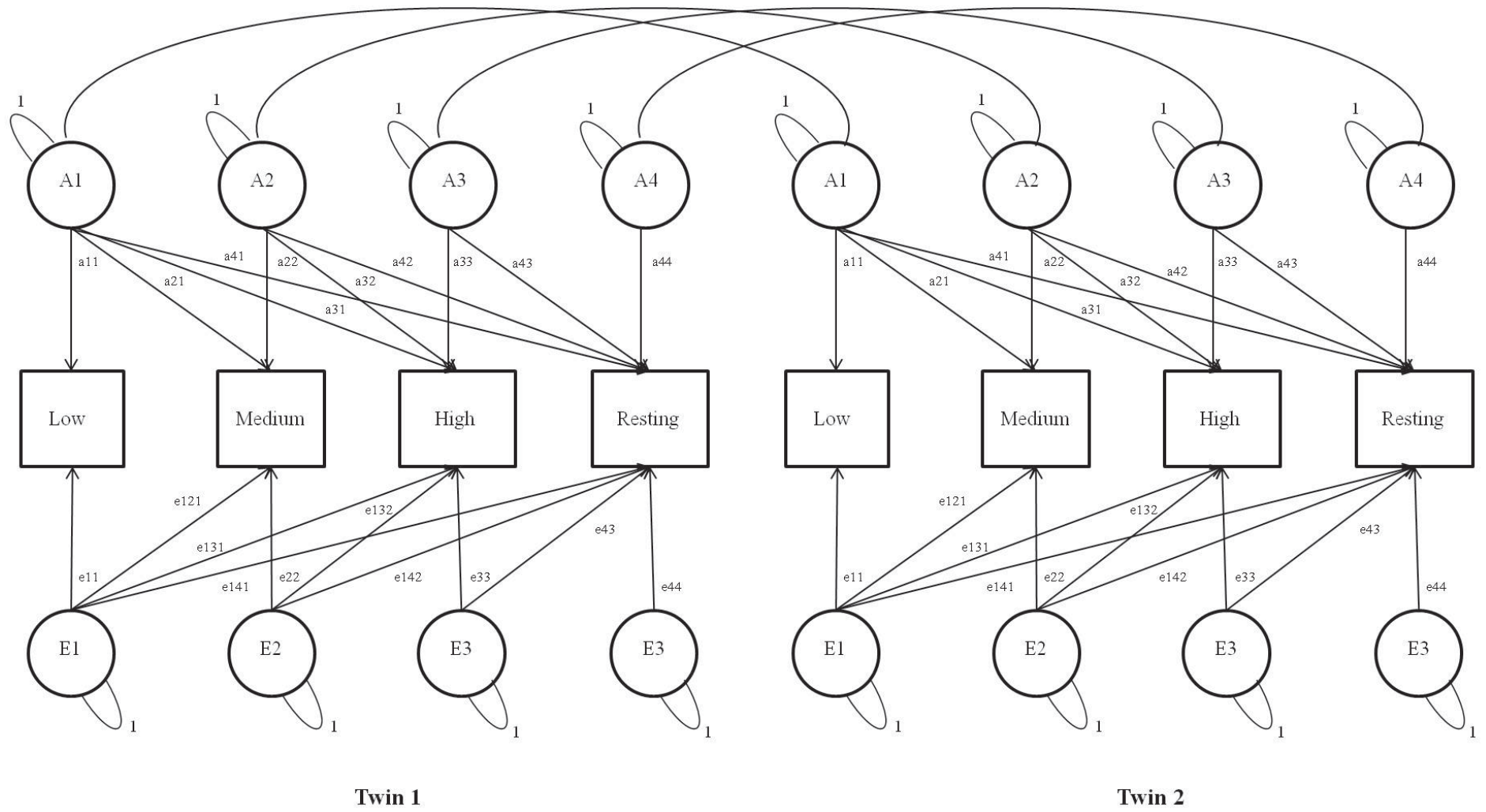

Supplement: Supplementary file 9 [file Image4.PDF]

**Supplemental Figure 5: Trivariate model**

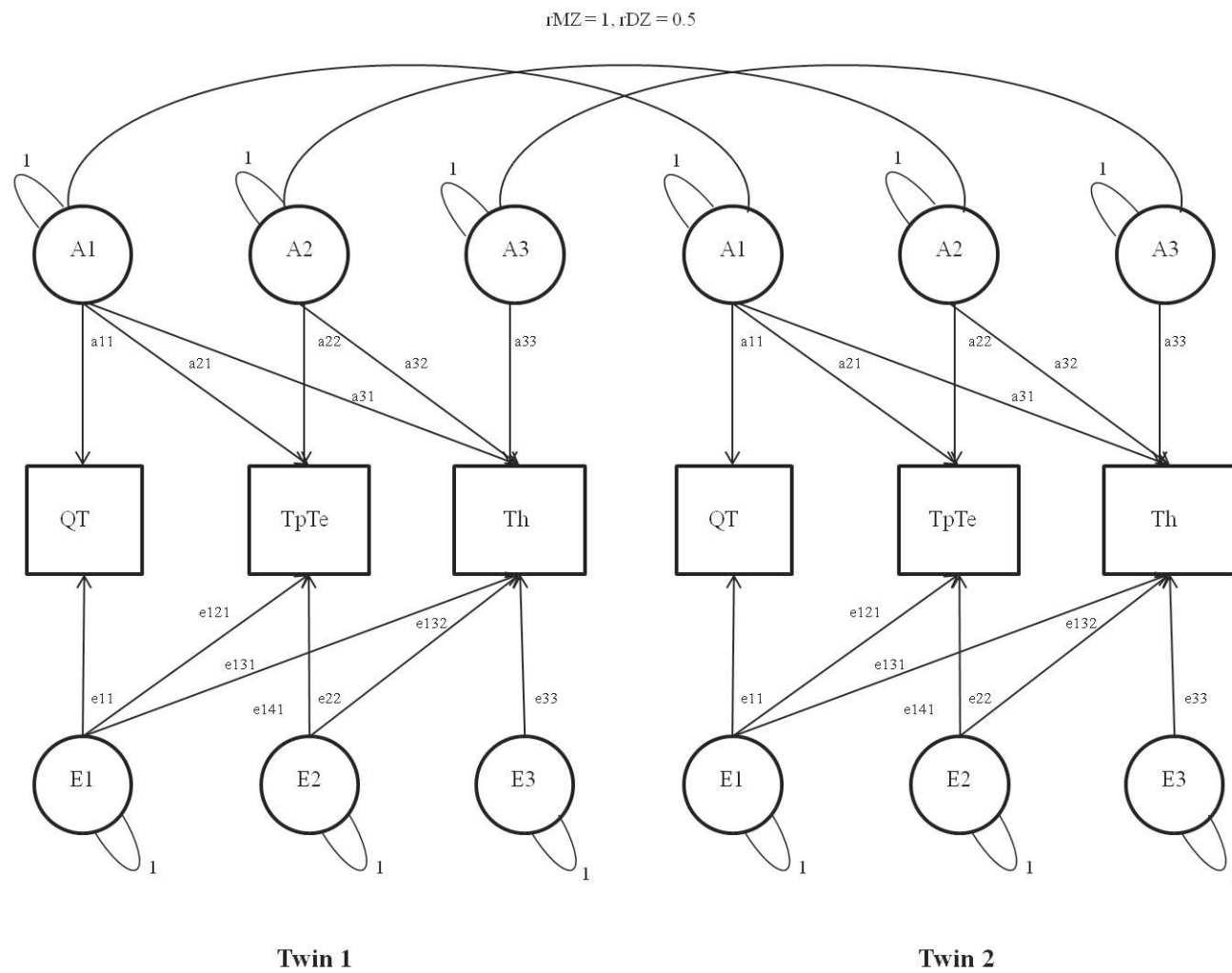

Supplement: Supplementary file 10 [file Image5.PDF]
