# Supplementary material for: Heritability of ECG Biomarkers in the Netherlands Twin Registry Measured from Holter ECGs
Source: Front Physiol. 2016 Apr 29;7:154. doi: 10.3389/fphys.2016.00154 (PMC4850154; doi:10.3389/fphys.2016.00154)
Supplement: Supplementary file 11 [file Image6.PDF]

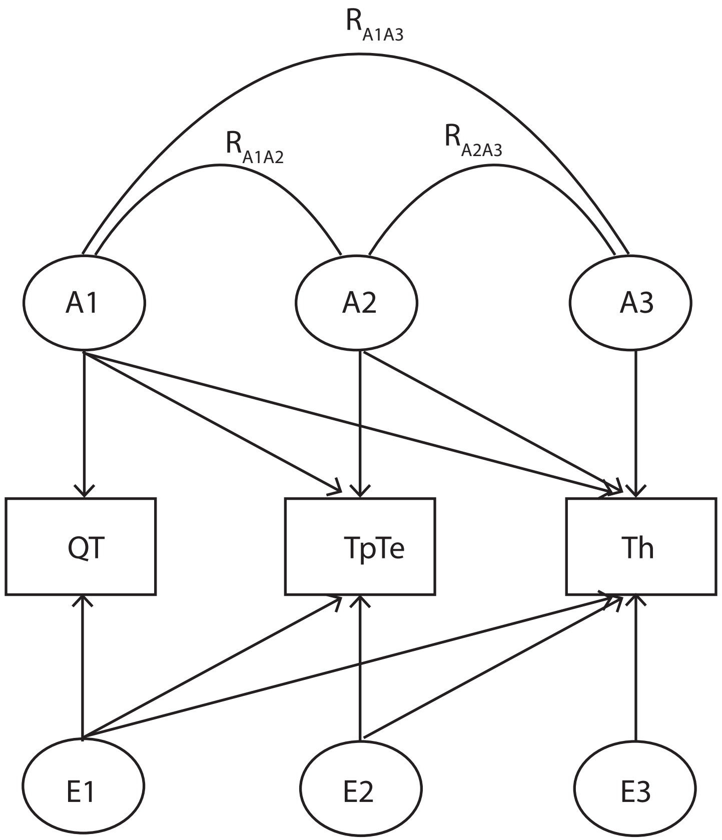

Supplemental Figure 6: Calculation of genetic correlations. An example of a trivariate model where observed traits (QT, TpTe and Th) are influenced by additove genetic factors (A1-A3) and unique envinromental factors (E1-E3). In this example, R_{A1A2} represents the genetic correlation between QT and TpTe, R_{A1A3} represents the genetic correlation between QT and Th, and R_{A2A3} represents the genetic correlation between TpTe and Th. The genetic correlation between QT and TpTe, for example, is calculated as follows: R_{A1A2} = covariance(A1, A2) / √ (variance(A1) * variance(A2)) and indicates to what extent the same genes are involved in the manifestation of both QT and TpTe.
